# Preoperative cognitive training improves postoperative cognitive function: a meta-analysis and systematic review of randomized controlled trials

**DOI:** 10.3389/fneur.2023.1293153

**Published:** 2024-01-08

**Authors:** Li Zhao, Yiping Guo, Xuelei Zhou, Wei Mao, Linji Li

**Affiliations:** ^1^Department of Anesthesiology, The Second Clinical Medical College, North Sichuan Medical College, Nanchong Central Hospital, Nanchong, China; ^2^School of Humanities and Management, Key Laboratory for Quality of Life and Psychological Assessment and Intervention, Guangdong Medical University, Dongguan, China; ^3^Nanchong Center for Disease Control and Prevention, Nanchong, China

**Keywords:** perioperative neurocognitive disorders, cognitive training, cognitive function training, postoperative cognitive dysfunction, postoperative delirium, meta-analysis

## Abstract

**Background:**

Postoperative cognitive dysfunction (POCD) and postoperative delirium (POD) are common post-surgical complications that often lead to prolonged hospitalization, reduced quality of life, increased healthcare costs, and increased patient mortality. We conducted a meta-analysis to evaluate the effects of preoperative cognitive function training on postoperative cognitive function.

**Methods:**

PubMed, Cochrane Library, Embase, Web of Science, ClinicalTrials, China National Knowledge Infrastructure, Wanfang Database, VIP Database, and Chinese Biomedical Literature Database were searched for randomized controlled trials comparing the effects of preoperative cognitive function training and conventional preoperative measures on postoperative cognitive function. The search period spanned from the establishment of the databases to March 31, 2023. The primary outcomes were the incidence of POCD and POD.

**Results:**

Eleven randomized controlled trials involving 1,045 patients were included. The results of the meta-analysis showed that, compared to the control group, preoperative cognitive function training significantly reduced the incidence of POCD (RR = 0.38, *P* < 0.00001), and there was no statistically significant difference in the incidence of POD (*P* = 0.3). Cognitive function training significantly improved postoperative cognitive function scores compared with the control group (MD = 1.92, *P* = 0.001). In addition, two studies reported that 10% of the patients in the cognitive training group completed a pre-set training duration.

**Conclusion:**

Cognitive function training significantly reduced the incidence of POCD; however, there was no significant difference in the incidence of POD. Preoperative cognitive function training should be promoted and emphasized as a simple, economical, and practical method of improving postoperative cognitive function.

**Systematic Review Registration:**

https://www.crd.york.ac.uk/PROSPERO/display_record.php?RecordID=396154

## 1 Introduction

Postoperative cognitive dysfunction (POCD) and postoperative delirium (POD) are common complications of surgical procedures that mainly manifest as cognitive dysfunction, especially learning, memory, and attention disorders (1–3). The incidence of POCD ranges from 30 to 41% (4) after non-cardiac surgery and is as high as 43% after cardiac surgery (5). The incidence of POD ranges from 4.7 to 13.7% (6). POCD and POD often result in patient and family suffering, including prolonged hospital stays, reduced quality of life, increased healthcare costs, and even increased mortality rates (7, 8). However, current research on the pathogeneses of POCD and POD is unclear and complex, and common risk factors include the patient's preoperative cognitive functional status, coexisting chronic diseases, nutritional status, anesthesia, surgery, and pain (9–11). Current evidence suggests that drug interventions have an uncertain effect on cognitive impairment (12, 13); therefore, non-drug interventions for POCD and POD can be an effective preventive measure and the focus of future research.

Cognitive function training is the most common non-pharmacological intervention for the clinical improvement of cognitive impairment (14). Cognitive function training refers to programs that encompass structured exercises targeting specific cognitive tasks, aiming to enhance performance in one or multiple cognitive domains, including, but not limited to, memory, attention, and executive function (15). Playing computer games, reading books, exercising, writing, practicing object or word recall, improving spatial memory, and promoting communication and interaction are among the most frequently employed methods of cognitive function training (16, 17). Cognitive function training significantly improves cognitive function in many cognitive disorders, including Parkinson's disease (18), stroke (19), mild cognitive impairment (20), and depression (21). However, whether preoperative cognitive function training can reduce the incidence of POCD and POD remains controversial. Saleh et al. found that preoperative cognitive function training significantly reduced the incidence of POCD after gastrointestinal surgery (16). However, O'Gara et al. found that preoperative and postoperative cognitive function training did not reduce the incidence of POCD or POD. In addition, the cognitive function training group demonstrated lower treatment adherence (22). There have been no meta-analyses of the effects of preoperative cognitive function training on POCD or POD. Therefore, we conducted a meta-analysis to investigate this topic.

## 2 Methods

### 2.1 Search strategy

This meta-analysis was performed following the guidelines outlined in the Preferred Reporting Items for Systematic Reviews and Meta-Analyses (PRISMA) checklist (23). This study was registered in PROSPERO under the number CRD4202339 6154 (crd.york.ac.uk/prospero/display_record.php?RecordID=396154). Two reviewers (Zhao Li and Guo Yiping) independently searched PubMed, Cochrane Library, Embase, Web of Science, ClinicalTrials, CNKI, Wanfang Database, VIP Database, and Chinese Biomedical Literature Database from the inception of the databases to March 31, 2023. The language used was limited to English or Chinese. The search terms used were as follows: “cognitive function training or cognitive training or cognitive intervention” and “postoperative cognitive dysfunction or POCD or postoperative delirium or POD.” In addition, we searched the reference lists of the identified articles for relevant studies and screened the International Clinical Trials Registry Platform.

### 2.2 Inclusion and exclusion criteria

The inclusion criteria were as follows: (1) Patients undergoing surgery under general anesthesia without cognitive impairment before surgery, regardless of age, sex, or type of surgery. (2) The experimental group underwent cognitive function training before surgery. (3) Patients in the control group were treated only for the disease itself without cognitive function training. (4) Outcome measures included the incidence of POCD and POD. (5) There was no statistical difference in cognitive function between the cognitive function training and control groups at the time of enrollment. (6) The included studies were randomized controlled trials. We excluded studies in which data could not be extracted or used for analysis.

### 2.3 Outcomes

The primary outcomes were the incidence of POD and POCD. The secondary outcomes were treatment compliance and scores of cognitive function.

### 2.4 Data extraction and assessment of risk of bias

Data extraction and quality assessment were performed independently by two authors (Zhao Li and Guo Yiping). Any disagreements were resolved through discussion with the corresponding author (Li Linji). The following information was extracted: the name of the first author, publication year, country, average age of the participants, sample size, type of surgery, and intervention measures. The study quality was assessed using the Cochrane Risk of Bias Tool. Data conversion tools were used to convert the interquartile ranges to means and standard deviations.

### 2.5 Statistical analysis

Data analysis was conducted using Stata (version 14) and Review Manager (version 5.3). Dichotomous and continuous data were statistically analyzed using the risk ratio (RR) and mean difference (MD) with a 95% confidence interval (CI). A significance level of *P* < 0.05 was considered statistically significant. Statistical heterogeneity was assessed using the *I*^2^ statistic, with *I*^2^ > 50% indicating high heterogeneity, and *I*^2^ < 50% indicating low heterogeneity. In case of high heterogeneity, a random-effects model was used, whereas a fixed-effects model was employed for low heterogeneity. Sensitivity and subgroup analyses were performed for studies with high levels of heterogeneity. Publication bias was evaluated using Egger's test and a funnel plot.

## 3 Results

### 3.1 Identification and characteristics of the studies

**Figure 1:**
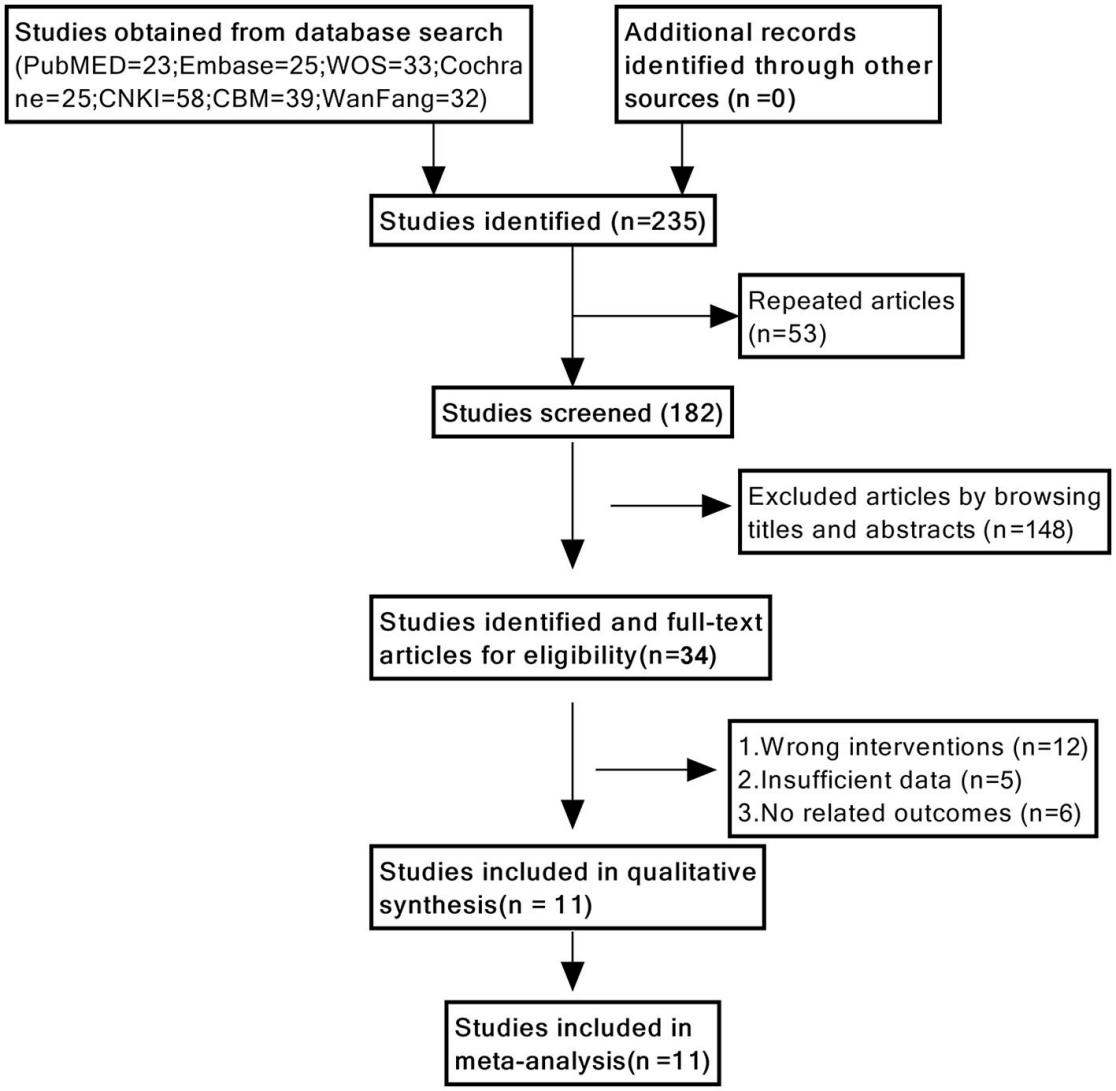
Flow diagram of the literature search strategy.

We initially identified 235 studies using a database search. Eventually, 11 studies were included (16, 17, 24–32), with a sample size of 1,045 cases, including 517 patients in the cognitive function training group and 528 patients in the control group. A flowchart of the study selection process is shown in Figure 1.

The characteristics of the studies are presented in Table 1. Eight and three studies assessed the effects of preoperative cognitive function training on POCD and POD, respectively.

**Table 1:** Characteristics of the included studies.

**References**	**Nation**	**Type of surgery**	**Outcome**	**Cognitive training group**				**Control group**		
				**Cases/total**	**Age (years)**	**Interventions**	**Preoperative training duration**	**Cases/total**	**Age (years)**	**Intervention**
Yi et al. (30)	China	Cardiac surgery	POCD within 10 days postoperatively	4/20	/	Knowledge learning	45 min per day for 7 days	9/20	/	Conventional treatment
Lei et al. (31)	China	Open surgery	POCD within 7 days postoperatively	7/45	67 ± 6	Memorizing spatial locations	Total 3 h 2 days before procedure	16/45	68 ± 6	Conventional treatment
Zhao (24)	China	Cardiac surgery	POCD within 7 days postoperatively	2/44	54.4 ± 5.7	Knowledge learning	2 h per day before surgery	17/45	54.4 ± 5.4	Conventional treatment
Saleh et al. (16)	China	Gastrointestinal tumor	POCD within 7 days postoperatively	11/69	71 ± 6	Memorizing spatial locations	3 h total	26/72	70 ± 6	Conventional treatment
Zhang et al. (25)	China	Urology	POCD within 1 day postoperatively	1/25	31 ± 8	Knowledge learning and mood guidance	1 day before surgery	5/25	30 ± 7	Conventional treatment
Guan and Longyan (26)	China	Spinal surgery	POD within 3 days postoperatively	2/30	71.2 ± 9.1	Memorizing necessities and mood guidance	30 min per day before surgery	13/30	69.4 ± 10.1	Conventional treatment
Wang and Ying (27)	China	Abdominal surgery	POCD within 7 days postoperatively	3/50	/	Memory and orientation training	Total 5 h 5 days before procedure	6/50	/	Conventional treatment
Li et al. (29)	China	Cardiac surgery	POCD within 14 days postoperatively	6/36	70.2 ± 2.5	Memory and orientation training	3 days before surgery	15/36	69.8 ± 1.8	Conventional treatment
Vlisides et al. (32)	USA	Non-cardiac non-neurosurgical	POD within 3 days postoperatively	6/23	66 ± 4.9	Playing computer games	20 min per day for 7 days	5/29	68 ± 5.4	Conventional treatment
Humeidan et al. (17)	USA	Non-cardiac non-neurosurgical	POD within 7 days postoperatively	18/125	67 (64–70)^#^	Playing tablet games	10 h total	29/126	67.5 (63–72)^#^	Conventional treatment
Tong and An-Ping (28)	China	Cardiac surgery	POCD within 7 days postoperatively	8/50	/	Memory and orientation training	Total 5 h 5 days before procedure	18/50	/	Conventional treatment

^#^Refers to median and interquartile spacing and the rest are means and standard deviations; “Knowledge learning” includes a detailed introduction of diseases, treatment measures, and learning of cognitive training methods; “Enhance communication” includes increasing communication with patients, improving the bad emotions of patients, etc. “cases/total” refers to the number of POCD or POD and the total number of people in the group.

### 3.2 Quality of the included studies

**Figure 2:**
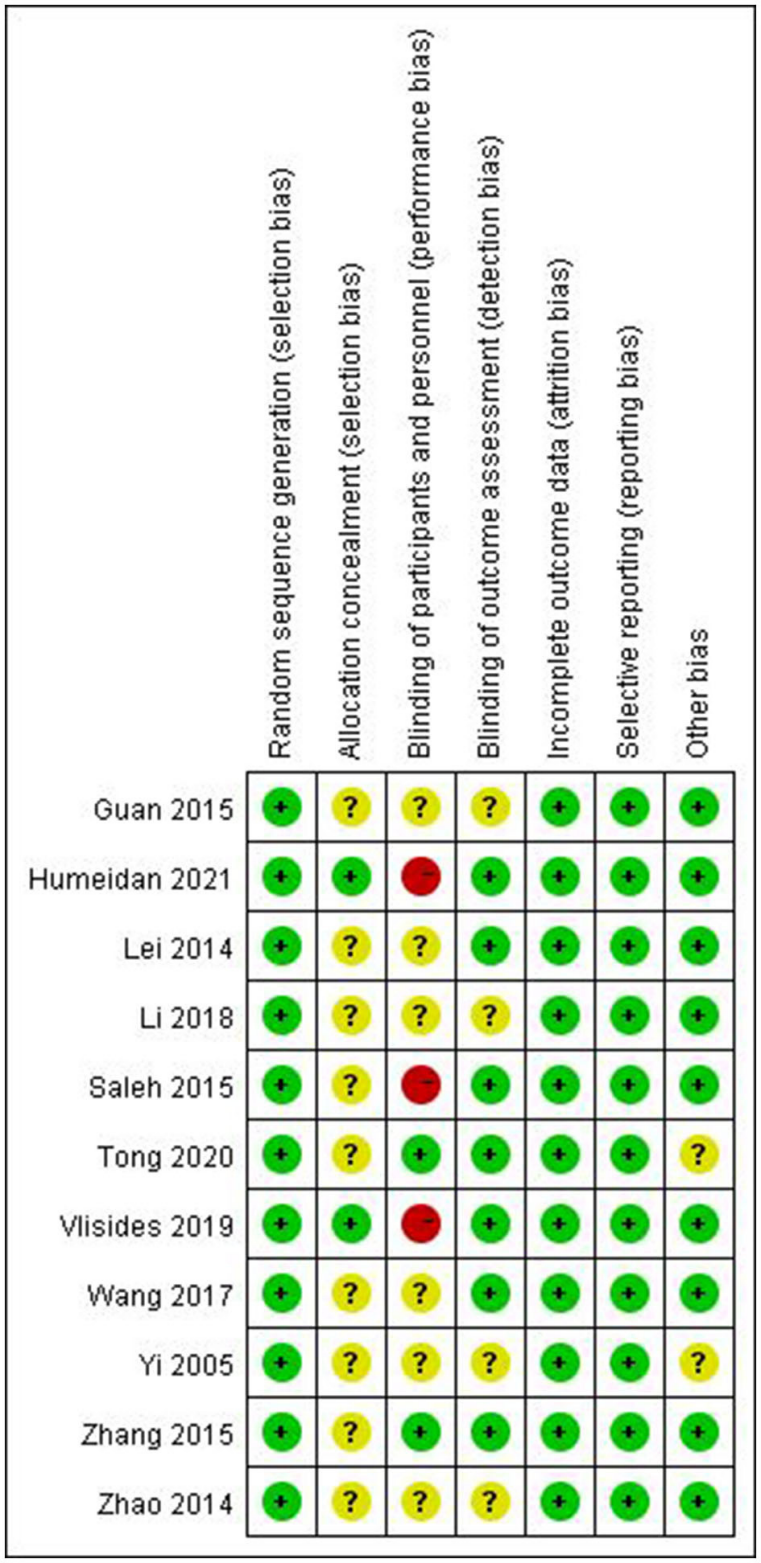
Risk-of-bias summary for all the trials.

The results of the risk of bias assessment for the included studies are shown in Figure 2. Nine studies were considered to have an unclear risk of allocation concealment (16, 24–31). Three studies (16, 17, 32) were considered to have high risk and six studies (24, 26, 27, 29–31) were considered to have an unclear high risk of participant blinding. Four studies (24, 26, 29, 30) were considered to have unclear risk in outcome assessment blinding. One study (28) did not provide a complete baseline value and another (30) had a small sample size, which may have contributed to other biases.

### 3.3 Primary outcome

#### 3.3.1 Incidence of POCD

**Figure 3:**
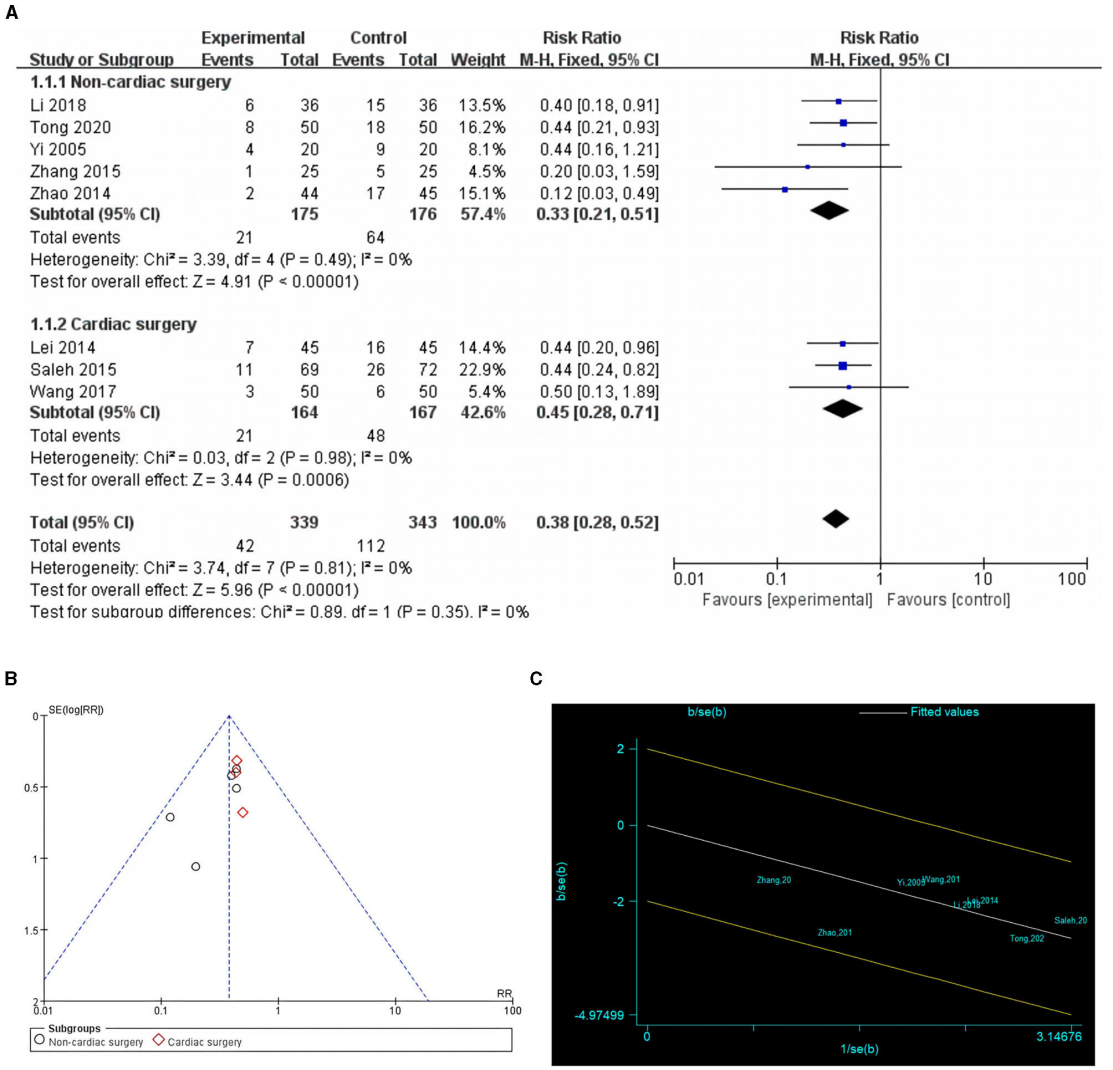
Comparison of POCD incidence in cognitive training and control groups. **(A)** Forest plot; **(B)** funnel plot; **(C)** Galbraith plots.

Eight studies with 682 patients assessed the incidence of POCD (16, 24, 25, 27–31). Five studies reported the incidence of POCD 7 days postoperatively (16, 24, 27, 28, 31). One study (25) reported POCD 1 day after surgery, one study (30) reported POCD 10 days after surgery, and another (29) POCD 14 days after surgery. In four studies (16, 25, 30, 31), POCD was defined when the neuropsychological test battery decreased by one standard deviation. In three studies (27, 28, 31) POCD was defined when Mini-Mental State Examination (MMSE) was < 24 points. One study (29) identified POCD as having a Montreal Cognitive Assessment (MoCA) score of < 26. Because of the low heterogeneity (*I*^2^ = 0%) and lack of significant heterogeneity in the Galbraith plot (Figure 3C), a fixed-effects model was used. The result showed that preoperative cognitive training significantly reduced the incidence of POCD compared with the control group (RR = 0.38, 95% CI: 0.28–0.52, *P* < 0.00001, Figure 3A). Egger's test (*P* = 0.098) and funnel plot (Figure 3B) did not reveal significant publication bias. Subgroup analysis showed that cognitive training significantly reduced the incidence of POCD in both cardiac and non-cardiac surgeries (*P* < 0.00001 and *P* = 0.0006, respectively).

#### 3.3.2 Incidence of POD

**Figure 4:**
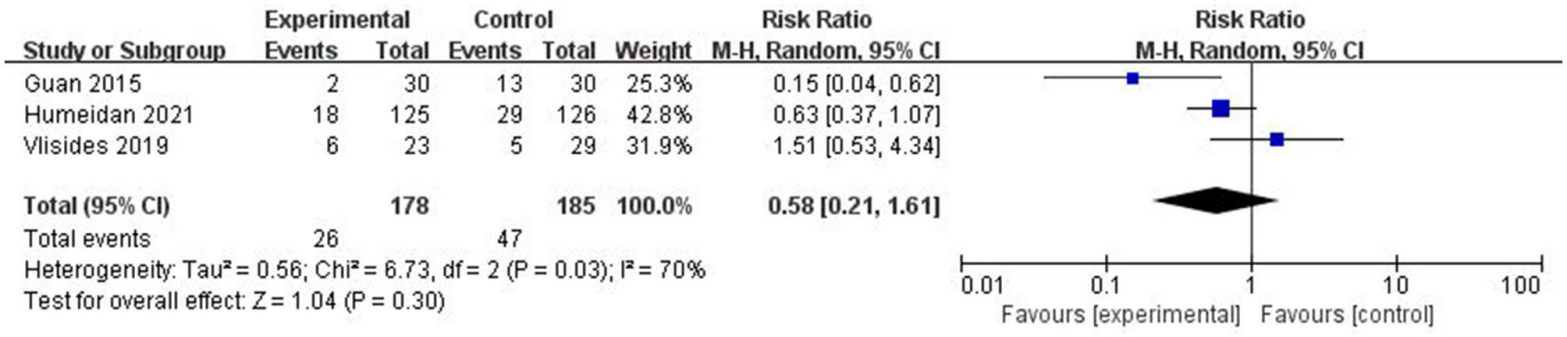
Forest plot of incidence of POD.

Three studies assessed the incidence of POD (17, 28, 32). Owing to the high heterogeneity (*I*^2^ = 70%), a random-effects model was used. There was no statistically significant difference between the two groups (RR = 0.58, *P* = 0.30; Figure 4). Sensitivity analysis and publication bias tests were not performed owing to the small amount of research literature.

### 3.4 Secondary outcome

#### 3.4.1 Scores of cognitive function

**Figure 5:**
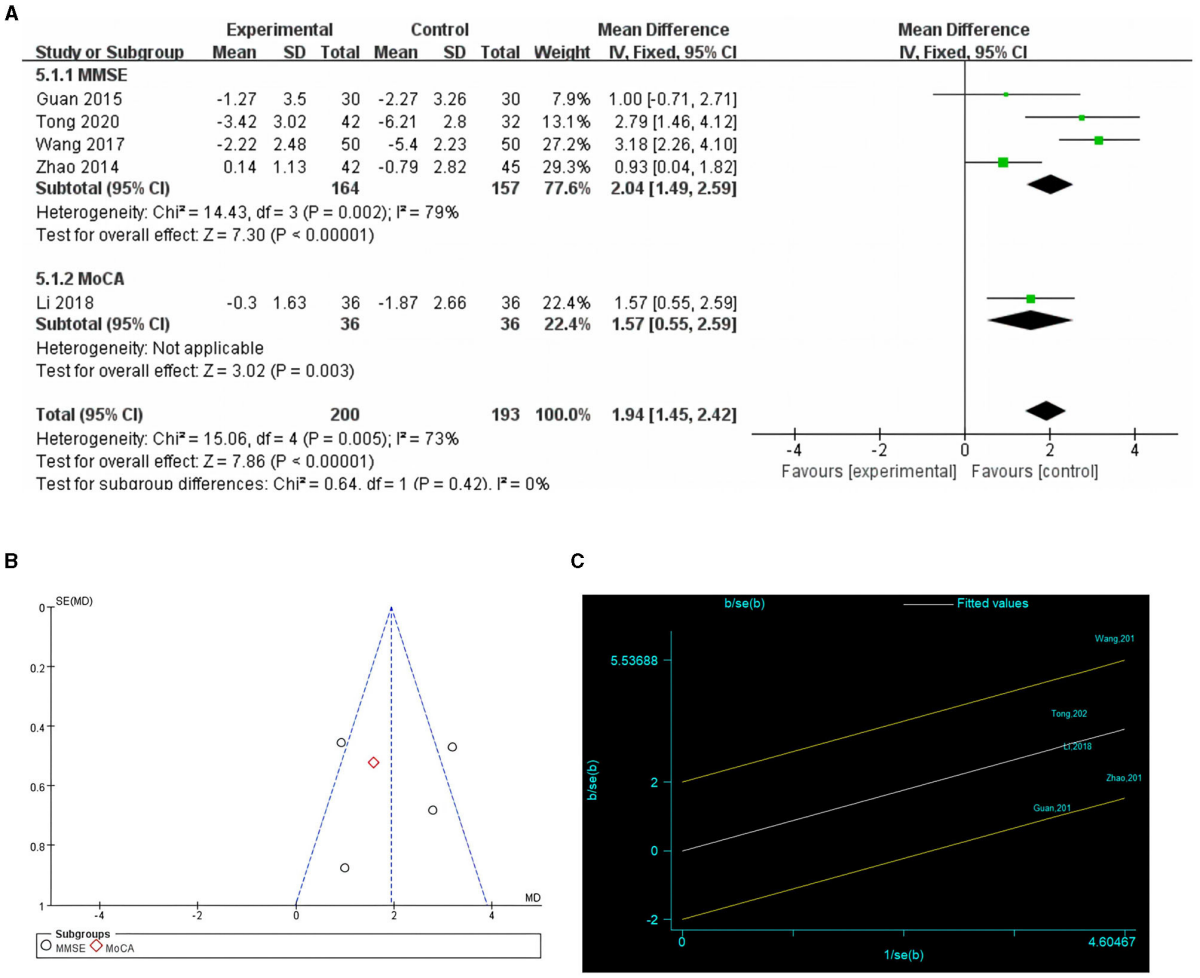
Comparison of cognitive function scores in cognitive training and control groups. **(A)** Forest plot; **(B)** funnel plot; **(C)** Galbraith plots.

Five studies reported postoperative cognitive function scores (24, 26–29), with four using the MMSE, and one using the MoCA (29). The difference between the postoperative cognitive function scores and pre-intervention baseline values was called the postoperative cognitive function score difference. This difference was used to indicate the degree of postoperative cognitive decline due to the effects of anesthesia and surgery. Owing to the high heterogeneity (*I*^2^ = 73%), a random-effects model was chosen. The results showed that preoperative cognitive function training significantly improved postoperative cognitive function scores compared to the control group (MD = 1.92, *P* = 0.0001, Figure 5A). Galbraith plots suggested that there was significant heterogeneity between the study by Wang et al. and the other studies (Figure 5C). Sensitivity analyses excluding the included studies one by one showed no significant changes in the results of the studies, indicating that the results were stable. The results of the subgroup analyses showed statistically significant differences between the results of the cognitive function training group and the control group in studies using the MMSE and MoCA scales (*P* = 0.002 and *P* = 0.003, respectively). Egger's test (*P* = 0.617) and funnel plot (Figure 5B) did not reveal significant publication bias.

#### 3.4.2 Treatment compliance of cognitive function training

**Figure 6:**
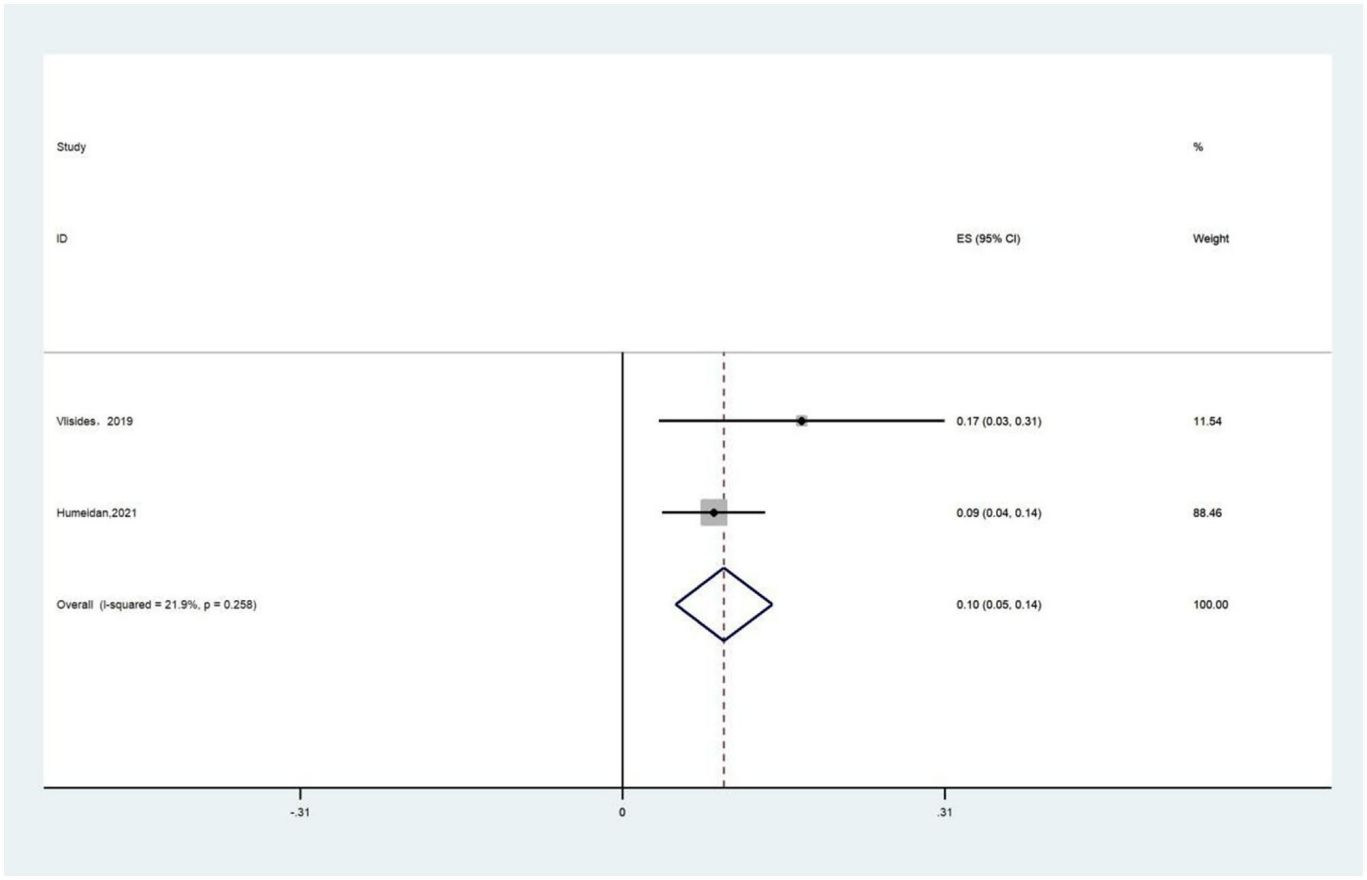
Forest plot of cognitive function training adherence.

Two studies reported treatment compliance of cognitive function training (17, 32). The proportion of patients who completed the predetermined training duration was referred to as treatment compliance. A fixed-effects model was chosen because of the presence of low heterogeneity (*I*^2^ = 21.9%) and the results showed that the completion rate of cognitive function training was 10% (Figure 6).

## 4 Discussion

As the demand for perioperative comfort treatment increases, more studies have begun to focus on perioperative neurocognitive disorders (PND) (33, 34). In this meta-analysis, we evaluated the effects of preoperative cognitive function training on postoperative neurocognitive function. This is the first meta-analysis to assess the effect of preoperative cognitive function training on POCD and POD.

The 2018 guidelines established a definition for PND that encompassed preoperative cognitive impairment, POD, delayed neurocognitive recovery, and cognitive decline identified within 2–12 months after surgery (35). As most previous studies have used POD and POCD as outcome indicators of postoperative cognitive function, we used POCD and POD to assess postoperative cognitive function.

This meta-analysis showed that preoperative cognitive function training significantly reduced the incidence of POCD compared to the control group (RR = 0.38, *P* < 0.00001), and the difference was not statistically significant in the incidence of POD (*P* = 0.30). Preoperative cognitive function training significantly improved postoperative cognitive function scores compared with the control group (MD = 1.92, *P* = 0.0001). In addition, treatment compliance with cognitive function training was 10%.

This meta-analysis showed that preoperative cognitive function training significantly reduced the incidence of POCD (*P* < 0.00001) with low heterogeneity (*I*^2^ = 0%) compared with conventional preoperative measures. The mechanism by which cognitive function training improves cognitive function may be as follows: first, cognitive function training may increase the density of cortical dopamine D1 receptors, which depend on adequate dopamine neurotransmission for key functions of human cognition (36, 37). Second, cognitive function training has been found to potentially enhance cognitive reserve. Some studies have found that patients with greater cognitive reserve, such as those with higher education, have a lower incidence of POCD (38). Therefore, cognitive function training may improve cognitive functions in patients by enhancing cognitive reserve (39). Third, cognitive function, perception, and memory function have been shown to decline progressively with age; however, the brain retains lifelong plasticity and adaptive reorganization ability. Therefore, some cognitive functions of the brain can be improved by using appropriately designed training programs (40, 41).

Anxiety is a risk factor of POCD (9). Since most studies did not achieve patient blinding, this may have led to some preoperative anxiety in patients in the preoperative routine treatment group compared to those in the cognitive training group, which may have biased the results. In most included studies, the control group did not receive any cognitive intervention. Assuming active control is used, such as a simple cognitive intervention. It can help in more accurately assessing the true effects of cognitive training and its cost-effectiveness ratio. Among the included studies, we assessed only the effects of preoperative cognitive training on postoperative cognitive outcomes. However, these studies lacked the transfer effect of preoperative cognitive training in everyday life or real-life scenarios. Future research could further explore the effects of cognitive training on real-life transfers and better understand the potential benefits of cognitive training in improving cognitive function.

This meta-analysis showed no statistically significant difference between the preoperative cognitive function training and control groups in terms of the incidence of POD (*P* = 0.30). The quality of evidence for this outcome metric was low, as only three studies reported POD.

Two studies reported a 10% rate of people completing the scheduled training duration (17, 32). One of these articles reported that the reasons for low adherence were lack of computer access, time constraints, and feeling overwhelmed (32). Therefore, simplification of training methods and provision of computer assistance are necessary to avoid low adherence to cognitive function training. As compliance was reported in only two studies, the quality of evidence for the outcome of treatment compliance was low. In addition, In O'Gara et al.'s study, it was found that most people completed >40% of their cognitive training duration (22).

Currently, various methods of cognitive training are available, including application software for electronic equipment, reading and writing training, mental activity, and compound cognitive training. More large-sample randomized controlled trials are needed to determine which of these training modalities is more helpful in improving postoperative cognitive function. A large number of current studies have used electronic devices for cognitive function training as a simple, economical, practical, and significant improvement in postoperative cognitive function, especially POCD, and should, therefore, be worthy of attention and promotion.

This meta-analysis revealed high heterogeneity in the pooled results. These heterogeneities may be due to differences in the duration and manner of cognitive function training. Furthermore, the diagnostic tools for cognitive function and the time of assessment were not consistent.

This study had some limitations. First, the sample size is relatively small. Second, some of the pooled results of this study suggest high heterogeneity. This heterogeneity may be due to differences in the duration and modality of cognitive function training, diagnostic tools for cognitive function, and inconsistencies in the timing of assessments. Third, because the incidence of POCD and POD was the primary outcome, we excluded studies that did not include data on POCD and POD; therefore, the application of secondary outcomes may be limited. Fourth, the main intervention in this study was preoperative cognitive function training; however, postoperative cognitive function training has also been reported to improve cognitive function (42). Fifth, the included studies only investigated the POCD incidence up to 14 days after surgery and lacked efficacy data beyond 1 month. Therefore, additional randomized controlled trials are required to verify the comprehensive effects of perioperative cognitive function training.

## 5 Conclusion

Our meta-analysis found that preoperative cognitive function training significantly reduced the incidence of POCD and improved postoperative cognitive function scores. However, the difference in the incidence of POD was not statistically significant. In addition, two studies showed low treatment compliance for cognitive function training. Preoperative cognitive function training should be promoted and emphasized as a simple, economical, and practical method of improving postoperative cognitive function.

## Data availability statement

The original contributions presented in the study are included in the article/supplementary material, further inquiries can be directed to the corresponding author.

## Author contributions

LZ: Writing—original draft, Writing—review & editing. YG: Writing—original draft, Writing—review & editing. XZ: Data curation, Formal analysis, Methodology, Software, Writing—original draft. WM: Data curation, Formal analysis, Methodology, Software, Writing—original draft. LL: Conceptualization, Investigation, Project administration, Supervision, Writing—review & editing.
